# Pemetrexed/cisplatin as first-line chemotherapy for advanced lung cancer with brain metastases: A case report and literature review

**DOI:** 10.1097/MD.0000000000004401

**Published:** 2016-08-12

**Authors:** Guangzhao He, Xiaoguang Xiao, Man Zou, Chengliang Zhang, Shu Xia

**Affiliations:** a Department of Pharmacy, Tongji Hospital of Tongji Medical College, Huazhong University of Science and Technology, Hankou, Wuhan; b Department of Pharmacy, Changzhou Tumor Hospital, Changzhou; c Department of Oncology, Tongji Hospital of Tongji Medical College, Huazhong University of Science and Technology, Hankou, Wuhan, P.R. China.

**Keywords:** brain metastases, cisplatin, first-line therapy, lung cancer, pemetrexed

## Abstract

**Background:**

Brain metastases (BMs) are a common and serious complication of non-small cell lung cancer (NSCLC). Whole-brain radiotherapy (WBRT), surgery, and molecular targeted therapy are usually used to treat NSCLC with BM. Chemotherapeutic options for BM are limited by tumor resistance, ineffective agents, and the blood–brain barrier. Pemetrexed/cisplatin is the preferred chemotherapy in nonsquamous NSCLC, but the efficacy of this treatment for nonsquamous NSCLC with BM is uncertain.

**Methods:**

We present a case of nonsquamous NSCLC with asymptomatic BM presenting with irritating cough and right shoulder back pain (unknown sensitizing epidermal growth factor receptor mutations or anaplastic lymphoma kinase).

**Results:**

He benefited from administration of first-line chemotherapy of pemetrexed/cisplatin. Partial remission was achieved in the primary lesion of the lungs and BM lesion. He was further given 3 cycles of pemetrexed monotherapy and WBRT. Complete remission was further achieved in BM lesion.

**Conclusion:**

The findings of clinical trials and theoretical studies about the current pemetrexed/cisplatin in the treatment of nonsquamous NSCLC with BM are also summarized to provide a reference for the application of pemetrexed/cisplatin in nonsquamous NSCLC with BM. Whether or not pemetrexed/cisplatin is definitely effective in nonsquamous NSCLC with BM must be proven by subsequent phase III clinical trials.

## 1 Introduction

Approximately, 10% of nonsquamous non-small cell lung cancer (NSCLC) cases with brain metastases (BMs) are definitely diagnosed at first visit.[ ^1^ ^2^] Aside from whole-brain radiotherapy (WBRT) and surgical treatment, targeted drugs (tyrosine kinase inhibitors/TKIs) are also used in nonsquamous NSCLC with BM. [^1^ ^2^ ^3^ ^4^ ^5^ ^6^] TKIs are recommended as first-line treatment for patients with positive sensitizing epidermal growth factor receptor (EGFR) mutations or anaplastic lymphoma kinase (ALK), whereas systemic chemotherapy and deferred WBRT should be considered in patients with asymptomatic BM regimen.[ ^7^ ^8^] However, the treatment of nonsquamous NSCLC with asymptomatic BM, especially with unavailable genotype, remains uncertain and mainly depends on patient's individual situation. This article presents a case with pemetrexed/cisplatin as the first-line therapy in nonsquamous NSCLC with BM, and reports that partial remission (PR) was achieved in the primary lung lesion, whereas complete remission (CR) was obtained in the BM lesion.

## 2 Case presentation

**Figure 1:**
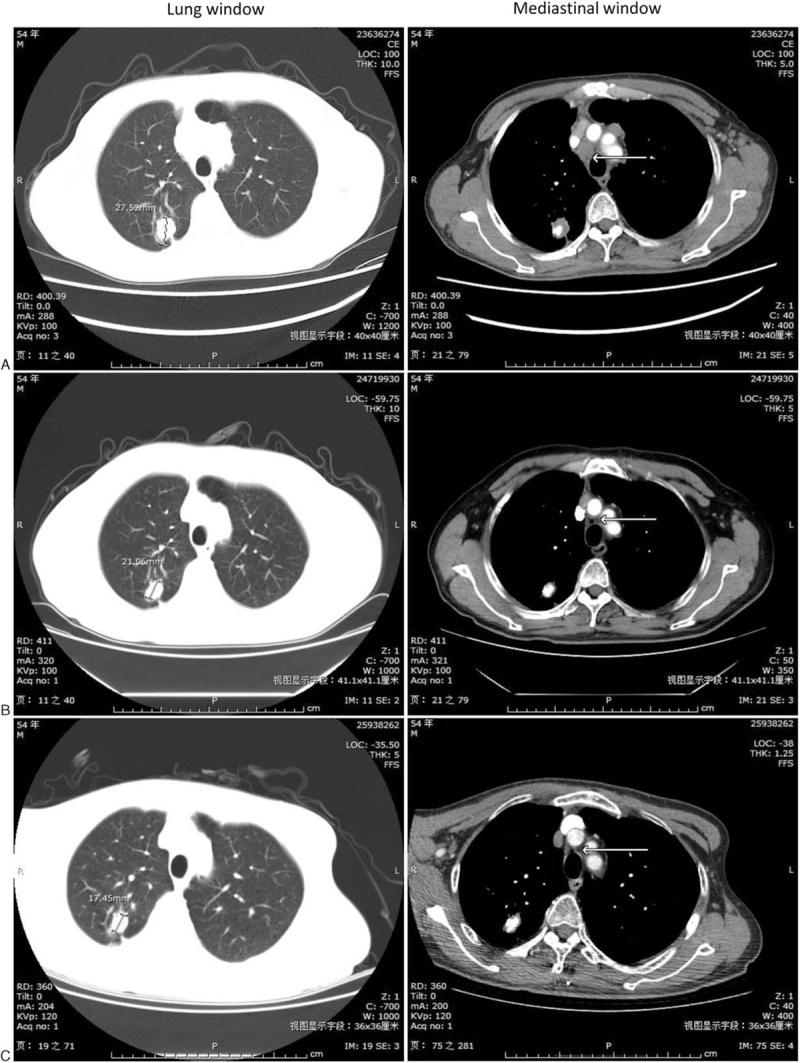
Image of chest computed tomography. (A) Before chemotherapy (November 2014); (B) After chemotherapy of pemetrexed/cisplatin for 6 cycles (April 2015); (C) After radiotherapy combined with chemotherapy of pemetrexed for 3 cycles (September 2015).

**Figure 2:**
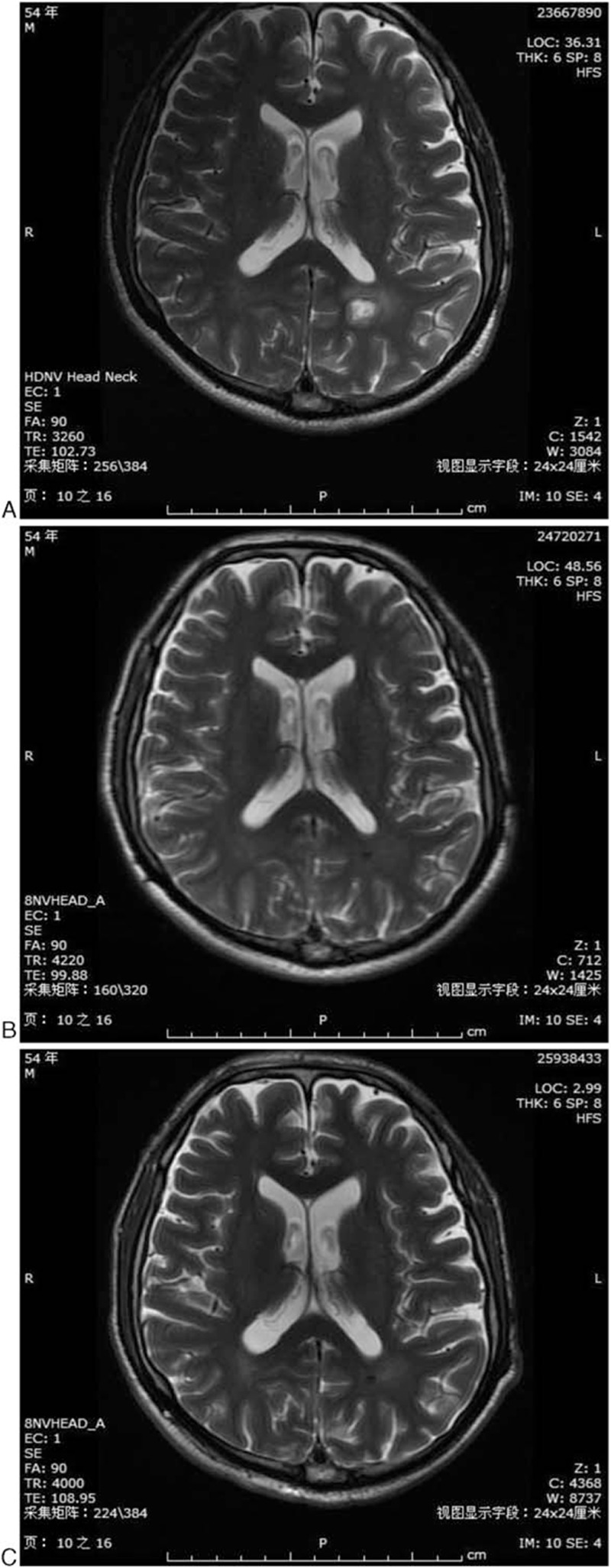
Image of brain magnetic resonance imaging. (A) Before chemotherapy (November 2014); (B) After chemotherapy of pemetrexed/cisplatin for 6 cycles (April 2015); (C) After radiotherapy combined with chemotherapy of pemetrexed for 3 cycles (September 2015).

The patient, a male former smoker (50 package-years) born in 1961, presented a medical history of hypertension. He presented with irritating cough without significant causes, accompanied by right shoulder back pain but without fever, headache, nausea, or vomiting. In November 2014, chest computed tomography (CT) revealed neoplastic nodules with calcification at the upper lobe of the right lung (28 mm × 19 mm), increased and enlarged lymph nodes at the bilateral hilum of the lungs, the mediastinum, the right cardiophrenic space, and the bilateral axillary fossa (Fig. 1A). Brain magnetic resonance imaging (MRI) suggested metastatic tumor as evidenced by the presence of nodular shadows at the left frontal lobe and at the posterior horn of the left ventricle (Fig. 2A). Aspiration cytology in the right cervical lymph nodes indicated metastatic adenocarcinoma. However, histological biopsy was strongly refused by the patient. The patient was finally diagnosed with stage IV lung adenocarcinoma (cT1N1M1) (unknown sensitizing EGFR mutations or ALK). He had quitted smoking since November 2014.

The patient was given 6 cycles of pemetrexed/cisplatin (pemetrexed 800 mg, cisplatin 120 mg, iv drip, day 1, every 3 weeks) from November 2014. Chest CT performed in April 2015 revealed that the right upper pulmonary nodular soft tissue and lymph nodes were significantly reduced (Fig. 1B) and brain MRI showed that only small pieces of hypointense were found in the left frontal lobe (Fig. 2B), which revealed PR in the primary lung lesion and BM lesion. The patient showed mild nausea, anorexia, and fatigue occurred after each cycle of chemotherapy. The patient was further given 3 cycles of pemetrexed monotherapy from April 2015 and WBRT in April 2015 (Dt30Gy/10F). Chest CT performed in September 2015 revealed stable disease (SD) (Fig. 1C), whereas brain MRI revealed CR (Fig. 2C). The patient showed dullness of mind and apathy for nearly 3 months. Maintenance therapy with oral gefitinib was used instead of pemetrexed from September 2015 because of the patient's strong refusal of further chemotherapy.

As of January 2016, the patient got >14 months of progression-free survival (PFS), PR for the lung lesion, and CR for brain lesion.

## 3 Discussion and literature review

In this case with asymptomatic BM, sensitizing EGFR mutations or ALK was unknown, so the evidence in choosing TKIs was insufficient while pemetrexed/cisplatin was selected as first therapy. Chest CT and brain MRI indicated that PR was achieved for lung cancer lesions and intracranial lesion after completion of 6 cycles of pemetrexed/cisplatin. The therapeutic effect of pemetrexed/cisplatin as the first-line therapy in nonsquamous NSCLC with BM was sufficiently demonstrated in this case, which excluded synergistic effects of TKIs, bevacizumab, and radiotherapy during these period, while not as reported by Liang et al. [^9^ ^10^ ^11^] Maintenance chemotherapy with pemetrexed monotherapy in parallel with WBRT or gefitinib was subsequently used to further consolidate the therapeutic effect.

Pemetrexed is a new antifolate chemotherapy drug, which exhibits an excellent antitumor activity against multiple malignant tumors including non-squamous NSCLC and malignant pleural mesothelioma. Pemetrexed/cisplatin was recommended as the first-line therapy for recurrent or progressed nonsquamous NSCLC.[ ^7^ ^8^ ^12^] Continuation maintenance of pemetrexed was also recommended in the absence of disease progression.[ ^7^ ^8^]

Several reports have indicated the potential benefits of pemetrexed on advanced nonsquamous NSCLC with BM. Ortuzar et al ^[6]^ retrospectively found that the incidence rate of advanced nonsquamous NSCLC with BM is significantly lower in the pemetrexed group than the non-pemetrexed group (docetaxel, gemcitabine) (3.0% vs. 7.3%, *P* < 0.001). Zhu et al ^[12]^ retrospectively found that patients of advanced non-quamous NSCLC with BM got median PFS of 5.0 months and a median overall survival (OS) of 11.0 months when received first-line pemetrexed/cisplatin.

One phase II trial evaluated first-line pemetrexed/cisplatin in asymptomatic nonsquamous NSCLC patients with BM. WBRT was given in case of disease progression (PD) during chemotherapy, or at completion of 6 cycles of chemotherapy. The study revealed intracranial, extracranial, and overall objective reaction rate of 41.9%, 34.9%, and 34.9% respectively, and median time to PD and OS of 4.0 and 7.4 months, respectively. ^[1]^ Another phase II trial evaluated pemetrexed/cisplatin parallel and synchronous with WBRT as the first-line therapy in nonsquamous NSCLC with BM. The study revealed intracranial, extracranial, and overall reaction rates of 68.3%, 34.1%, and 36.6%, respectively, and median PFS and OS of 10.6 and 12.6 months, respectively. ^[2]^ Both studies suggested that pemetrexed/cisplatin was an effective and tolerable first-line therapy for nonsquamous NSCLC with BM.

Multiple phase III clinical trials of pemetrexed as the first-line therapy for nonsquamous NSCLC with BM are currently underway. Current evidences of WBRT, pemetrexed, and bevacizumab for nonsquamous NSCLC with BM were integrated in the NCT02162537, and first-line WBRT + pemetrexed/cisplatin ± bevacizumab was compared with pemetrexed/cisplatin ± bevacizumab + WBRT (only performed after the progress of BM on the basis of clinical and imaging evidence). One of the inclusion criteria for the clinical trial was the patients should be negative for EGFR mutation or unavailable EGFR mutation.[ ^5^ ^13^] Considering that gefitinib and pemetrexed can be effectively used in nonsquamous NSCLC with BM, the study of NCT01951469 investigated the curative effect and safety of monotherapy with gefitinib as well as gefitinib combined with pemetrexed/cisplatin for nonsquamous NSCLC with BM.[ ^14^ ^15^] Previous studies reported that monotherapy with pemetrexed is effective for nonsquamous NSCLC with BM and that the drug concentration is too low in cerebrospinal fluid (CSF) under normal dosage; hence, NCT02284490 investigated the curative effect of monotherapy with high-dose pemetrexed (900 g/m^2^) for lung adenocarcinoma with BM.[ ^16^ ^17^] Although the efficacy of pemetrexed/cisplatin for nonsquamous NSCLC with BM has been proven by multiple pieces of evidence, clinical benefits of this regimen are still limited. On the basis of the current achievements of clinical trials, combination with WBRT, bevacizumab, and TKIs or high-dose pemetrexed is expected to evolve the primary method for treating nonsquamous NSCLC with BM.

Most chemotherapy drugs are inefficient in treating NSCLC with BM because of their difficulty in passing through the blood–brain barrier (BBB). ^[18]^ The mechanism of pemetrexed used in NSCLC with BM was investigated primarily from the perspective of pemetrexed passing through the BBB. Stapleton et al ^[19]^ investigated the pharmacokinetics of pemetrexed in plasma and CSF after the venous administration of pemetrexed in non-human primates and found that the concentration of pemetrexed in CSF is lower (0.33%–1.58%) than the drug concentration of pemetrexed by 2%. The study suggests that pemetrexed could have a very limited capability to pass through the normal BBB. Kumthekar et al ^[20]^ investigated the pharmacokinetics of pemetrexed in plasma and CSF in patients with BM or leptomeningeal metastases and reported that the concentrations of pemetrexed in CSF are lower than the drug concentrations of pemetrexed by 5% within 1 to 4 days after pemetrexed administration. Similarly, the study suggested very limited capacity of pemetrexed to pass through the normal BBB of patients with BM or leptomeningeal metastases. Other studies on the mechanism by which pemetrexed passes through the BBB are currently lacking. Dogan et al ^[21]^ hypothesized the following mechanisms by which pemetrexed passes through the BBB in nonsquamous NSCLC patients with BM: the permeability of the BBB could be changed by pretreatment with dexamethasone or dehydration treatment with mannitol before pemetrexed administration; secretion of bradykinin might be affected by pemetrexed, or the permeability of cerebral vessels is increased because of the possible pemetrexed-induced upregulation of bradykinin B2 receptor; the low expression of P-glycoprotein in new vessels of lesions of NSCLC with BM facilitates the passage of the drug through the BBB. The above theoretical presumptions need to be proven by further studies. Similar with pemetrexed, the mean peak CSF/plasma total cisplatin ratio was about 3% followed by intravenous infusion in patients with the solid type of metastatic brain tumor. ^[22]^ Whether or not the capability of pemetrexed or cisplatin to bypass the BBB is increased by WBRT also warrants further investigation. Also, we should mention that systemic response rates were roughly similar to intracranial response rates suggesting that BBB may be overestimated as a major impediment to efficacy. ^[23]^ Whether there are other factors affecting the sensibility of BM lesion to chemotherapy needs to be further evaluated.

In summary, when sensitizing EGFR mutation and ALK are positive, TKIs maybe a reasonable choice for advanced NSCLC with BM.[ ^7^ ^24^ ^25^] While pemetrexed/cisplatin as the first-line chemotherapy for advanced lung cancer with BM maybe suitable for asymptomatic BM with negative or unknown EGFR mutation and ALK.

## 4 Conclusions

Our case has been demonstrated as having the outstanding efficacy of pemetrexed/cisplatin in nonsquamous NSCLC with asymptomatic BM. Whether or not pemetrexed/cisplatin is definitely effective in nonsquamous NSCLC with BM should be further proven by subsequent phase III clinical trials.
